# Trends in Mortality Rates from Cardiovascular Disease and Cancer between 2000 and 2015 in the Most Populous Capital Cities of the Five Regions of Brazil

**DOI:** 10.36660/abc.20180304

**Published:** 2020-02

**Authors:** Wolney de Andrade Martins, Maria Luiza Garcia Rosa, Ricardo Cardoso de Matos, Willian Douglas de Souza Silva, Erito Marques de Souza Filho, Antonio José Lagoeiro Jorge, Mario Luiz Ribeiro, Eduardo Nani Silva

**Affiliations:** 1 Universidade Federal Fluminense - Programa de Pós-Graduação em Ciências Cardiovasculares, Niterói, RJ - Brazil; 2 Universidade Federal Fluminense - Departamento de Medicina Clínica, Niterói, RJ - Brazil; 3 Universidade Federal Rural do Rio de Janeiro (UFRRJ), Seropédica, RJ - Brazil

**Keywords:** Cardiovascular Diseases/mortality, Coronary Artery Diseases/physiopathology, Neoplasms/mortality, Epidemiology

## Abstract

**Background:**

In many cities around the world, the mortality rate from cancer (CA) has exceeded that from disease of the circulatory system (DCS).

**Objectives:**

To compare the mortality curves from DCS and CA in the most populous capital cities of the five regions of Brazil.

**Methods:**

Data of mortality rates from DCS and CA between 2000 and 2015 were collected from the Mortality Information System of Manaus, Salvador, Goiania, Sao Paulo and Curitiba, and categorized by age range into early (30-69 years) and late (≥ 70 years), and by gender of the individuals. Chapters II and IX of the International Classification of Diseases-10 were used for the analysis of causes of deaths. The Joinpoint regression model was used to assess the tendency of the estimated annual percentage change of mortality rate, and the Monte Carlo permutation test was used to detect when changes occurred. Statistical significance was set at 5%.

**Results:**

There was a consistent decrease in early and late mortality from DCS in both genders in the cities studied, except for late mortality in men in Manaus. There was a tendency of decrease of mortality rates from CA in São Paulo and Curitiba, and of increase in the rates from CA in Goiania. In Salvador, there was a decrease in early mortality from CA in men and women and an increase in late mortality in both genders.

**Conclusion:**

There was a progressive and marked decrease in the mortality rate from DCS and a maintenance or slight increase in CA mortality in the five capital cities studied. These phenomena may lead to the intersection of the curves, with predominance of mortality from CA (old and new cases).

## Introduction

Cardio-oncology has emerged as a new area of study and practice, resulting from numerous epidemiological and clinical interactions between diseases of the circulatory system (DCS) and cancer (CA). This interrelationship is supported by the prevalence of common risk factors, population aging, advances in diagnostic and treatment techniques, and cardiovascular injuries secondary to CA treatment.

One of the common questions in cardio-oncology is where the intersection point between the curves of mortality for DCS and CA will be, i.e., when DCS will become the leading cause of mortality thereafter.^1^ Circulatory diseases have become the most prevalent causes of death in Brazil, followed by CA, since the decrease in the prevalence of infectious diseases.^2,3^ In developed countries, there has been a fall in the mortality from DCS since the mid-1960s,^4,5^ and deaths from CA outweigh deaths from DCS.^6^ In Brazil, there has been a reduction in the rate of mortality from DCS since the 1980s, for both sexes, especially in the South and Southeast regions.^7^ Concomitantly with this trend, the number of deaths due to CA in Brazil has grown; it went from the fifth to the third cause of death from 1980 to 2000, and today, CA is the second cause of mortality.^8^

Cancer is the leading cause of death in half of the United States of America (USA) and in some Western European countries. It has a close relationship with population aging. The drop in mortality from DCS is partly attributed to improved diagnosis and treatment.^9-11^ However, both DCS and CA have a complex relationship mediated by several risk factors common to both, like smoking and alcoholism, overweight and obesity, eating pattern, sedentary lifestyle; hypertension, and diabetes mellitus.^12,13^

There are few studies that seek to understand the relationship between CA and DCS in the Brazilian population. Patterns of morbidity and mortality in Brazil have changed over the years. Demographic and epidemiological transitions, differences in access to health care, genetic peculiarities, among other factors, have resulted in the formation of regional population groups with particular characteristics.^14^ Analysis of temporal trends in mortality based on population data could further clarify the scenario.

This study aimed to compare early and late mortality, by gender, from CAD and CA between 2000 and 2015 in the most populous capital cities of each Brazilian region and in the country as a whole.

## Methods

It was decided to study the mortality rates from DCS and CA in the most populous capital cities, one of each of the five federated states of Brazil. The Federal District was not included. Demographic and mortality data were obtained from the Brazilian Institute of Geography and Statistics (IBGE). The following cities were included: Manaus (Northern region), São Paulo (Southeast), Goiania (Central west), Curitiba (South), and Salvador (Northeast).

Early and late mortality was defined using the age ranges of 30-69 years and ≥70 years, according to the definition of early mortality from non-communicable diseases, stratified by gender, adopted by the Brazilian Ministry of Health, and in line with the recommendations published in the United Nations’ World Population Prospects.^14^

Data of mortality from 2000 to 2015 were obtained from the Department of Informatics of the Brazilian Unified Health System (DATASUS), and following the International Classification of Diseases (ICD)-10 as follows: mortality from DCS (chapter IX of ICD-10) and specific causes - acute rheumatic fever and chronic rheumatic heart diseases (066), hypertensive diseases (067), ischemic heart diseases (068), acute myocardial infarction (068.1), other forms of heart diseases (069), cerebrovascular diseases (070), atherosclerosis (071), and other and unspecified disorders of the circulatory system (072), and mortality from neoplasms (chapter II of ICD-10) and specific causes: malignant neoplasms, lip, oral cavity and pharynx (032), malignant neoplasms of esophagus (033), malignant neoplasms of stomach (034), malignant neoplasms of colon, rectum and anus (035), malignant neoplasms of liver and intrahepatic bile ducts (036), malignant neoplasms of pancreas (037), malignant neoplasms of larynx (038), malignant neoplasms of trachea, bronchi and lungs (039), malignant neoplasms, skin (040), malignant neoplasm of breast (041), malignant neoplasm of cervix uteri (042), malignant neoplasm of corpus uteri and uterus, part unspecified (043), malignant neoplasm of ovary (044), malignant neoplasm of prostate (045), malignant neoplasm of bladder (046), malignant neoplasm of meninges, brain and other parts of the central nervous system (047), non-Hodgkin's lymphoma (048), multiple myeloma and malignant plasma cell neoplasms (049), leukemia (050), in situ neoplasms, benign neoplasms and neoplasms of uncertain behavior (051), and other malignant neoplasms (052).

The data were obtained from the computerized databases of the death certificate records of the Brazilian Mortality Information System (Vital Statistics System) and “Population estimates: city, gender and age 2000-2015 RIPSA IBGE” (division of Demographic and Socioeconomic sector). All the information was collected from the DATASUS website.^15^

### Statistical analysis

To assess the tendency of the estimated annual percentage change (EAPC) of the mortality rate from DCS and CA during the study period, the Joinpoint regression model (joinpoint software version 4.6.0.0 National Cancer Institute, Bethesda, Maryland, EUA)^16^ was used. The Monte Carlo permutation test was used to detect the years when significant changes in the trends occurred.^17,18^ Also, the Poisson distribution was used with the JoinPoint regression model. Assuming such distribution, a maximum of two joinpoints were selected. The software calculates the annual percentage change by the parametric method, with a 95% confidence interval for each segment of trend. The program calculated adjusted mortality rates for sex and age using the standard population based on WHO 2000-2025. Statistical significance was set at 5%.

## Results

**Figure 1:**
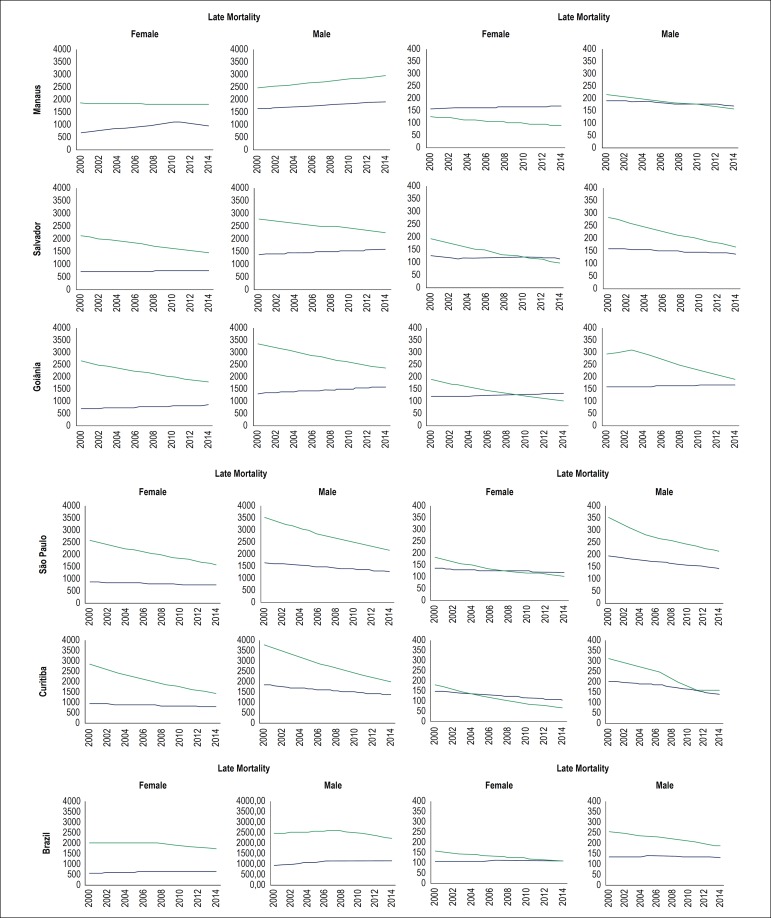
Trends in mortality rates from cardiovascular disease and cancer in the most populated capital cities of five Brazilian regions, stratified by gender and age group, 2000-2015. Source: DATASUS. Green curve represents cardiovascular diseases and blue curve represents cancer

Figure 1 shows the curves of mortality from DCS and CA in the five capitals studied. For all groups and age ranges, there was a decrease in DCS, more pronounced in early mortality, especially in women, among whom CA is already the leading cause of mortality. A stability trend or a slight increase in mortality rate was found in the CA curves. In late mortality, there was a striking difference between the rates of mortality from DCS and CA, with higher rates of deaths from DCS compared with deaths from CA. Thus, the intersection point of these curves occurs later as compared with early mortality.

Table 1 shows the EAPCs in mortality from DCS and CA in the most populous capital cities of the five regions of Brazil between 2000 and 2015. Between 2000 and 2015, there was a consistent decrease in early and late mortality from DCS, in both sexes, in the most populous capitals, except for late mortality in men in the city of Manaus. The EAPC for mortality from DCS ranged from -6.5% for early mortality in women in São Paulo, to 0.3%, for early mortality among men in Curitiba. This variation, however, was not statistically significant, probably resulted from an inversion of the trend, with increased mortality in the last two years analyzed, i.e., 2014 and 2015. The reductions were comparable between genders and more pronounced in early mortality. Interestingly, the coefficients of late mortality from DCS were at least ten times the coefficients of early mortality.

**Table 1:** Trends in estimated annual percentage change of mortality from diseases of the circulatory system and cancer in the most populated capital cities of the five geographic regions of Brazil, 2015

Capitals		Early mortality				Late mortality			
		Male		Female		Male		Female	
		CA	DCS	CA	DCS	CA	DCS	CA	DCS
Manaus	APC 95%CI	-0.8* (-1.5;0)	-2.0* (-2.9;-1.1)	0.5 (-0.4;1.3)	-2.3* (-2.8;-1.7)	1.1* (0.5;1.7)	1.1* (0.5;1.7)	-3.0 (-6.6;0.8)	-0.3 (-0.9;0.3)
Salvador	APC 95%CI	-0.9* (-1.4;-0.4)	-3.4* (-4.1;-2.7)	-1.7 (-4.4;1.1)	-4.3* (-4.9;-3.6)	0.9* (0.3;1.5)	-1.4* (-2.1;-0.8)	0.5 (0;1)	-2.4* (-2.9;-1.9)
Goiania	APC 95%CI	0.4 (-0.4;1.2)	-4.0* (-4.5;-3.4)	0.7* (0.1;1.4)	-4.0* (-4.8;-3.2)	1.2* (0.4;2)	-2.3* (-3;-1.7)	1.4* (0.7-2.1)	-2.5* (-3;-2)
São Paulo	APC 95%CI	-1.9* (-2.1;-1.7)	-2.6* (-3.1;-2.2)	-0.9* (-1.1;-0.8)	-2.9* (-3.5;-2.3)	-1.5* (-1.7;-1.3)	-3.1* (-3.3;-2.8)	-1.0* (-1.3;-0.8)	-3.1* (-3.4;-2.8)
Curitiba	APC 95%CI	-3.4* (-4.2;-2.7)	0.3 (-6.8;7.8)	-2.2* (-2.8;-1.5)	-6.5* (-7.1;-5.9)	-1.9* (-2.3;-1.4)	-4.1* (-4.5;-3.6)	-1.1* (-1.9;-0.3)	-4.4* (-4.8;-3.9)
Brazil	APC 95%CI	-0.9* (-1.1;-0.7)	-2.7* (-3.5;-1.9)	0.1 (-0.1;0.4)	-2.4* (-2.6;-2.2)	0.1 (-0.1;0.3)	-2.1* (-2.5;-1.6)	0.2 (-0.1;0.4)	-2.1* (-2.6;-1.7)

* indicates statistically significant association (p < 0.05); CA: cancer; DCS: diseases of the circulatory system; EAPC: estimated annual percentage change; CI: confidence interval

Differently from DCS, the coefficients of mortality from CA showed different behaviors by regions, time periods and genders. In general, there was a decreasing trend in the rates of mortality from CA in São Paulo and Curitiba in all time periods and sexes. In contrast, there was an increase in the rates of mortality from CA in all periods in Goiania. In Salvador, there was a mixed behavior, characterized by a drop in early mortality in men and women and increment in late mortality in both genders.

Manaus was a major exception in terms of mortality rate behavior; mortality from DCS decreased in all time periods except for the late mortality in men. On the other hand, early mortality from CA exceeded that from DCS in both men and women.

Therefore, the determinant of the convergence of the mortality curves seems to be the greater decrease in mortality from CD.

Table 2 shows the intersection points (years), real or presumed, of the mortality curves from CA and DCS. Regarding early mortality, CA ranks first in males in Goiania, and in women in all the studied cities. In late mortality, however, the intersection of the curves has not occurred in any of the cities yet, and may occur, it he trends continue, from 2026 on.

**Table 2:** Estimated intersection point (year) of the mortality curves from diseases of the circulatory system and cancer in the most populated capital cities of the five geographic regions of Brazil

Locality	Age range of early mortality		Age range of late mortality	
	Male	Female	Male	Female
	Year of intersection			
Manaus	2009	1992	-	-
Salvador	2023	-	2031	2038
Goiania	2018	2000	2026	2034
São Paulo	2071	2009	2047	2051
Curitiba	-	2004	2032	2033
Brazil	2035	2015	2045	2057

Source: DATASUS, according to chapters II and IX from International Classification of Diseases-10; age of early mortality: between 30 and 60 years old; age of late mortality: older than 70 years

Table 3 shows the three main causes of mortality, stratified by early and late, and by gender, in the most populous capital cities of the five regions. There were differences between the cities, the age ranges and the genders.

**Table 3:** Three main causes of specific deaths (according to the International Classification of Diseases-10) in the most populated capital cities of the five geographic regions of Brazil, 2015

Gender/Age range	Manaus		Salvador		Goiania		São Paulo		Curitiba	
	Cancer	DCS	Cancer	DCS	Cancer	DCS	Cancer	DCS	Cancer	DCS
Female 30-69 years	Cervix	CVD	Breast	CVD	Breast	IHD	Breast	IHD	Breast	IHD
	Breast	IHD	Colon	IHD	Lung	CVD	Lung	CVD	Lung	CVD
	Lung	MI	Lung	MI	Cervix	MI	Colon	MI	Colon	MI
Female ≥ 70 years	Lung	CVD	Breast	CVD	Breast	CVD	Breast	IHD	Breast	CVD
	Cervix	IHD	Colon	IHD	Colon	IHD	Colon	CVD	Colon	IHD
	Breast	MI	Lung	MI	Lung	HD	Lung	MI	Lung	MI
Male 30-69 years	Stomach	IHD	Lung	IHD	Lung	IHD	Lung	IHD	Lung	IHD
	Lung	CVD	Prostate	CVD	Colon	MI	Colon	MI	Colon	MI
	Larynx	MI	Pharynx	MI	Pharynx	CVD	Stomach	CVD	Stomach	CVD
Male ≥ 70 years	Prostate	CVD	Prostate	CVD	Prostate	CVD	Prostate	IHD	Prostate	CVD
	Lung	IHD	Lung	IHD	Lung	IHD	Lung	MI	Colon	IHD
	Stomach	MI	Colon	MI	Colon	MI	Colon	CVD	Lung	MI

Source: DATASUS. DCS: diseases of the circulatory system; CVD: cerebrovascular disease; IHD: ischemic heart diseases; MI: myocardial infarction; HD: hypertensive diseases; for the analysis, MI was considered a separate cause (from ischemic diseases) of death, and the sections 069 (chapter about DCS) and section 052 (chapter about cancer) of the International Classification of Diseases-10 were not included in the ranking of diseases

## Discussion

### Mortality from DCS and CA in Brazil compared with the world

In Brazil, the discussion about the increase in cancer mortality is more recent than in European countries and USA, where the epidemiological transition occurred earlier than in Brazil. In Brazil in 2005, 32% of deaths were caused by DCS, followed by cancer (15%). At that time, Rosa et al.^19^ drew attention to a probable intersection of the curves of mortality from DCS and CA. In the United Kingdom, in 2011, the DCS passed from the first cause of mortality to second position for the first time since the middle of the 20^th^ century;^20^ 29% of the deaths were caused by CA, while 28% by DCS.^21^ The reduction of mortality from DCS in the United Kingdom was explained by a decrease in the mortality from myocardial infarction, increase of pharmacological and surgical treatments, and decrease of risk factors like smoking.^21-23^ Similar situation to Brazil was observed in the USA, where mortality from DCS decreased more than from CA. If this tendency continues, CA will be the leading cause of deaths in 2020.^24^

The different stages of growth and development of the Brazilian regions made us make a particularized analysis, since it is difficult to draw a reliable picture of Brazil as a whole. The choice of the most populous capitals came from the assumption of a higher degree of urbanization and its influence on the health of the inhabitants. In general, in the western world, the interception of the mortality curves is caused by a marked decrease of mortality from DCS, especially in more developed countries in terms of socioeconomic development.

### General trend of the curves of mortality from DCS and CA in Brazil

Analysis of the historical trend of the curves of mortality from DCS and CA reveled an important and sustained decrease of deaths from DCS in the most populous capital cities of each of the five Brazilian regions, except for Manaus. In this city, late mortality from DCS increased in men. Data from Brazil showed that DCS continue the main cause of mortality. However, an analysis of the cities revealed that CA already surpassed DCS as the leading cause of deaths in nearly 10% of the Brazilian cities.^25^

The results of the present work suggest two patterns of trends that led to the grouping of the five capitals into two subgroups: in the first subgroup, São Paulo and Curitiba, whose pattern is more similar to that of developed countries, i.e., with a significant fall in mortality from DCS, plus maintenance or slight decrease of mortality from CA. In this pattern, convergence of the curves results from the decrease in deaths from DCS. In the second pattern, Goiania, Salvador and Manaus, where there was also a decrease in mortality due to DCS, but less significant, in contrast to a modest increase in mortality from CA. In this second group, the convergence of the curves takes longer to occur. Manaus showed a singular behavior, with increase of late mortality from DCS in males.

In the Brazilian cities studied, data of 2015 showed that ischemic heart disease and cerebrovascular disease were the main causes of DCS. While individuals in the early age group die more from ischemic heart disease, at late age, mortality from cerebrovascular disease is higher. Between 1996 and 2011, in Brazil, there was a consistent decrease in mortality rate due to cerebrovascular disease in both genders, with differences in the magnitude of decrease between the regions.^26^ In addition to socioeconomic development, the control of cardiovascular risk factors and a considerable increase (450%) in the access to primary care services, may have contributed to the decrease.^27^ As observed in developed countries, efforts to diagnosis and treatment of risk factors and comorbidities have probably contributed to the decrease of stroke mortality,^28^ and hence to the decrease of mortality from DCS.

### Regional trends in the curves of mortality from DCS and CA

São Paulo and Curitiba presented a decrease in early and late mortality in both genders. It could be partly explained by the greater access to the diagnosis and treatment of CA. Chemotherapy and radiotherapy services are more concentrated in the Southern and Southeastern regions of Brazil.^29^

In Salvador, it was observed a decrease in early mortality from CA in men. Lung cancer has a high lethality and is the main type of cancer in this population. It is currently the main cause of death among men in North America and Europe and its mortality has significantly increased in Asia, Latin America and Africa.^30^ In Brazil, adenocarcinoma is the main cause of early mortality among men and is related to the high prevalence of smoking in male sex.^15,31^ The decrease in CA mortality in Salvador can be attributed to the public policies for CA prevention during the last decades, and in 2004, Salvador presented the lowest smoking rate in Brazil.^32^ On the other hand, late mortality from CA has increased among men and women. One hypothesis for such increase among women is the high mortality rates from breast cancer, which represents the leading cause of late mortality in women.^15^ Mortality rates from breast cancer in the Brazilian population have shown geographic variations, with a trend to stabilization in the southeast, decline in the south and increase in the north, northeast and central-west regions. In the northeast region, between 2000 and 2010, CA mortality increased by 100% in white women, a population subgroup that increased by only 10% in size in the period. In contrast, there was a 183% increase in mortality among black women, with a respective population growth by 58%.^33^ A possible explanation for the increase in late mortality rates from CA among men in Salvador is the high percentage of Afro-Brazilians living in this city. Prostate cancer is the leading cause of late mortality among men in Brazil^15^ and a black man has 1.6 of being diagnosed and 2.4 higher odds of dying from prostate cancer than a white man.^34^

Manaus greatly differs from the other capital cities regarding the pattern of the mortality curves from DCS and CA. Early mortality from CA significantly increased among women. Cervical cancer is the main type of cancer,^15^ whose mortality rates increased in the north and northeast regions.^35-37^ In Manaus, early mortality decreased whereas late mortality increased among men. The main causes of early death from CA was gastric cancer followed by lung cancer. In Brazil, mortality rates from gastric cancer significantly increased in individuals older than 59 years. In the north region, mortality rates have increased in individuals of both sexes older than 75 years.^37^ Prostate cancer mortality continues to increase in Brazil, with a vast number of under-reported or late-diagnosed cases.

In Goiania, early mortality and late mortality from CA increased in both men and women. Early death from CA was mainly caused by breast CA in women. This may be explained by the difficult access to appropriate diagnosis, since only 18% of the mammography machines available in the whole State of Goias belong to the Unified Health System, and 80% of the population living in the state are users of the public health system.^38,39^ Another possible reason is the fact that mammograms is performed at relatively late age in Goiania, 49 years old.^40^

### Differences between sexes in the trends of mortality from DCS and CA

Early and late mortality rates from DCS were lower in women than in men in all studied capitals and showed a more marked decrease over the years among women than men. One hypothesis for these findings is the fact that women are more adherent to primary healthcare programs for the screening and prevention of diseases.

With respect to CA, regional differences were found in the incidence of different tumors of varying mortality rates. For example, in Sao Paulo, colon CA ranks the second in incidence and cervical CA is in the fourth position, whereas in Manaus, cervical cancer ranks the first.^37^

Breast CA is the most prevalent cancer among women in Brazil and in most of the studied capitals. The treatment may include surgery, chemotherapy, radiotherapy, and hormonal therapy. Despite many advances in the treatment of breast CA, such as the use of immunohistochemical tests and anti-HER-2 agents, the access to these therapies by users of the health public system occurred later, and probably had no effect on the outcome of the patients included in this research.

Some limitations need to be considered when analyzing the results of this study. Data analyzed in this study were obtained from death certificates, and hence subject to inaccuracy. The diagnosis of CA is confirmed by imaging tests and/or anatomopathological examination, which confer greater reliability. The diagnoses of DCS are essentially established by clinical examination. Also, it is worth mentioning that the results were obtained from populations living in large urban centers; extrapolations to medium- and small-sized cities may not be appropriate, as reproducibility of these data is not necessarily guaranteed. Finally, determinants of mortality and estimates of trends can be influenced by public polices.

## Conclusion

In general, and considering specific regional exceptions, there was a gradual and marked decrease in mortality rates from DCS in the five Brazilian capital cities studied, whereas mortality rates from CA remained unchanged or showed a slight increase from 2000 to 2015. Such events will lead to the intersection of the mortality curves, with perspective of a predominance of CA (old and new cases) mortality.
